# Cytologic Comparison Between Malignant and Regenerative Nodules in the Background of Cirrhosis

**DOI:** 10.5812/hepatmon.5954

**Published:** 2012-07-30

**Authors:** Bita Geramizadeh, Najma Asadi, Seyed Ziyaodin Tabei

**Affiliations:** 1 Department of Pathology, Shiraz University of Medical Sciences, Shiraz, IR Iran; 2 Transplant Research Center, Shiraz University of Medical Sciences, Shiraz, IR Iran

**Keywords:** Carcinoma, Hepatocellular, Liver Cirrhosis, Biopsy, Fine-Needle

## Abstract

**Background:**

Incidence of hepatocellular carcinoma has been increased as the sixth most common cancer in the world. Improvement in imaging techniques has decreased the need for tissue confirmation in diagnosis of hepatocellular carcinoma (HCC). Meanwhile, false negative and positive cases are present. Fine needle aspiration (FNA) biopsy can be helpful to identify well-differentiated HCCs with low risk of vascular invasion and better prognosis following transplantation.

**Objectives:**

We conducted this study to find useful criteria for cytological differential diagnosis between nodules of well differentiated hepatocellular in the background of cirrhosis and pure cirrhotic regenerative nodules in cytology smears.

**Materials and Methods:**

140 fine needle aspirations (FNA) of fresh cirrhotic hepatectomy specimens were studied (100 pure regenerative nodules and 40 HCC nodules). All slides were reviewed by two expert pathologists. The most useful criteria were selected and evaluated in 560 cytology smears stained by Pap and Wright methods.

**Results:**

None of the smears from cirrhotic patients showed mitosis, transgressing endothelium, eccentric nuclei, and scant cytoplasm, but thick nuclear membrane, spindle cells and abundant, thick and monotonous cytoplasm were found in many cases with cirrhosis. Large nucleoli (2 %), multiple nucleoli (6 %), increased N/C ratio (4 %), and broad cores (2 %) were found very rarely in the smears of regenerative nodules, but they were present in 50 %, 72.5 %, 87 %, and 77.5 % of HCC nodules, respectively.

**Conclusions:**

Combination of cytologic criteria can be helpful for differential diagnosis between HCC and regenerative nodules.

## 1. Background

Hepatocellular carcinoma (HCC) is the sixth most common malignancy in the world and the third cause of cancer deaths [1]. Differential diagnosis between regenerative cirrhotic nodules and hepatocellular carcinoma (HCC) is challenging both in imaging and cytological studies. In spite of good performances of radiologic criteria, especially CT scan, in diagnosis of HCC, the classic combination of signs consisting of hypervascularity and portal washout is neither perfectly sensitive nor entirely specific [1]. In regard to liver needle biopsy, the risks of needle tract seeding and haematogenous dissemination have been actively introduced [2]. Hepatocellular carcinoma (HCC) represents a major epidemiological problem in Europe, the USA and developing countries, so percutaneous FNA cytology represents one of the best methods for obtaining diagnostic material in patients suspected to HCC in terms of cost-effectiveness, and individual preference and expertise [3]. Fine needle aspiration (FNA) is a known and popular technique for evaluation of nodules in the patients with diagnosis of cirrhosis, because liver cirrhosis is known to predispose hepatocellular carcinoma. However, in cytology smears, sometimes it can be difficult to differentiate regenerative nodules of cirrhosis from well differentiated HCC because of similarities between tumor cells and benign hepatocytes [4][5]. There are many studies documenting various criteria for this cytological differential diagnosis, but problem of distinguishing well differentiated HCC from regenerative nodules still exists. It is mostly due to the limited number of cirrhotic livers which have been aspirated [4][5]. On the other hand, current recommendations include immediate work up of HCC nodules with large diameter and more frequent screening of smaller nodules, because early detection of HCC in patients with cirrhosis can improve the patient’s survival and successful treatment [6].

## 2. Objectives

In this study we performed fine needle aspiration (FNA) in 140 fresh unfixed explanted cirrhotic livers received in pathology lab (100 regenerative nodules of pure cirrhosis and 40 cirrhosis with HCC nodules) to find out the most valuable criteria for differential diagnosis.

## 3. Materials and Methods

**Figure 1:**
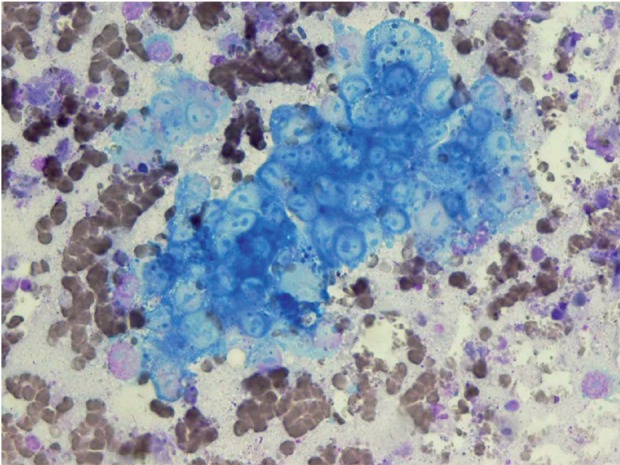
Macro Nucleoli in a Cluster of Malignant Cell (Wright stain X 250)

**Figure 2:**
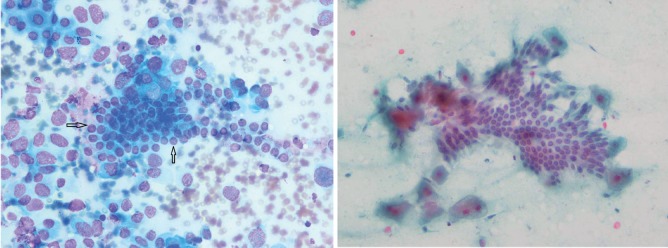
Groups of Bile Duct Epithelial Cells. A: Wright Stain X250, B: Pp stain X250

**Figure 3:**
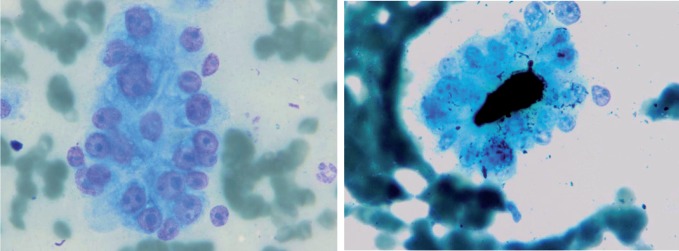
Acinar Transformation. A: without Bile in the center (Wright stain X 250), B: With Bile in The Center (Wright stain X 250)

**Figure 4:**
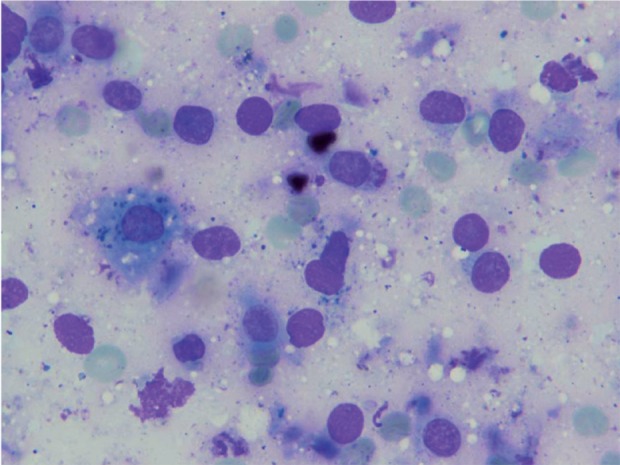
Many Atypical Naked Nuclei (Wright stain X 250)

FNA was performed in 100 fresh explanted livers with cirrhosis without HCC nodules (61 males and 39 females, mean age 30.1 ± 15.8). The etiology of cirrhosis was hepatitis B in 87 % and hepatitis C in 13 % of the patients. FNA was also performed in HCC nodules of 40 fresh explanted livers (20 males and 20 females, mean age 33 ± 10.7). Etiology of cirrhosis in this group was hepatitis B in 38 cases and combined hepatitis B and C in two patients. All of the FNAs were performed by 22 gauge needle through multiple passes. Two air-dried and two alcohol fixed smears were prepared and stained by Wright and Pap methods, respectively. The location of the FNA was accurately identified and a histologic section was taken for further histological diagnosis (Hematoxylin and Eosin) and confirmation. We applied various cytologic criteria in both groups. Main applied criteria were as below: Cytoplasmic findings (scant and abundant cytoplasm, thick cytoplasm, and monotonous cytoplasm), Nuclear findings (thick nuclear membrane, eccentric nuclei, large nucleoli defined as easily visible at X 250 , multiple nucleoli defined as more than two nucleoli (Figure 1), and irregular chromatin pattern), high N/C ratio (more than 1/4), transgressing endothelium (endothelial cells which cause connections between large clusters), endothelial wrapping, broad cords defined as cords with more than three cells thickness, presence of spindle cells, mitosis, bile duct epithelial cells (Figure 2) , pseudoacini formation (Figure 3A, B), and naked nuclei (tendency to dissociation) narrow cords (Figure 4). It is worthy to note that the 560 slides from these 140 cases were reviewed by two senior pathologists who were blind about final diagnosis. All of HCC nodules in the background of cirrhosis were histologically confirmed and immunohistochemistry was performed for Hep-Par-1.

## 4. Results

In Table 1 the frequencies of cytological findings in two groups of cirrhotic patients with and without HCC were shown in details. None of cirrhotic cases without HCC showed small cell dysplasia but large cell dysplasia was present in 33 % of the cases. Small cell dysplasia was also commonly identified in cirrhotic background of HCC cases. All of cirrhotic smears showed hepatocytes with abundant thick cytoplasm. (Table 1) . None of cirrhotic patients showed mitosis, transgressing endothelium, eccentric nuclei and scant cytoplasm, but thick nuclear membrane, spindle cells and abundant, thick and monotonous cytoplasm were found in many cirrhotic cases. Large nucleoli (2 %), multiple nucleoli (6 %), increased N/C ratio (4 %), and broad cords (2 %) were observed very rarely in regenerative nodules, but they were present in 50 %, 72.5 %, 87 %, and 77.5 % of HCC nodules, respectively. We didn’t find endothelial wrapping in any of HCC and regenerative nodules.

**Table 1:** Details of Frequencies of Cytological Findings in Two Groups of Cirrhotic Patients With and Without Hepatocellular Carcinoma

	**‍Cirrhosis, No. (%)**	**Hepatocellular Carcinoma , No. (%)**	**P value**
Thick Cytoplasm	100 (100)	16 (40)	< 0.001
Abundant cytoplasm	100 (100)	13 (32.5)	< 0.001
Monotonous Cytoplasm	99 (99)	16 (40)	< 0.001
Mitosis	0	8 (20)	< 0.001
Transgressing endothelium	0	4 (10)	< 0.006
Eccentric nuclei	0	14 (35)	< 0.001
Scant cytoplasm	0	27 (67.5)	< 0.001
Large nucleoli	2 (2)	20 (50)	< 0.001
Multiple nucleoli	6 (6)	29 (72.5)	< 0.001
Broad cords	2 (2)	31 (77.5)	< 0.03
Increased N/C ratio	4 (4)	35 (87)	< 0.001
Irregular chromatin pattern	2 (2)	31 (77)	< 0.001
Atypical naked nuclei	35 (35)	30 (75)	< 0.001
Spindle cells	82 (82)	8 (19)	< 0.001

## 5. Discussion

Cirrhotic patients need surveillance to pick up small suspicious nodules by imaging [7]. Cohort studies indicate that surveillance of patients with cirrhosis might lead to a better prognosis by treatments such as resection, ablation, and liver transplantation, but these approaches are more effective for smaller tumors [8]. Therefore it seems reasonable to attempt to early detection of HCC in patients with cirrhosis [6]. FNA has been introduced as an accurate method for the diagnosis of HCC in patients with cirrhosis [7], but there are few studies comparing cytomorphological findings of macroregenartive nodules of cirrhosis with well differentiated HCC, most of which have been on the limited number of cirrhotic regenerative nodules[4]. So, we decided to perform FNA cytology in fresh explanted livers after hepatectomy for orthotropic liver transplantation. We tried to do this procedure as similar as possible to ultrasound and CT scan guided FNA by 22 gauge needles [9]. We selected 100 hepatectomy specimens with pure cirrhosis secondary to hepatitis B and C and 40 explanted livers with cirrhosis secondary to hepatitis B and C with nodules of HCC to compare cytologic findings and to select the most discriminatory criteria for better differential diagnosis. We used all the cytologic criteria that have been proposed in the previous literature to find out the best and most accurate criteria for distinction between HCC and regenerative nodules [2][4]. None of the cirrhotic cases without HCC showed mitosis, transgressing endothelium, eccentric nuclei and scant cytoplasm, but these criteria were found in 20 %, 10 %, 35 %, and 67.5 % of HCC nodules, respectively. Therefore although these findings are not very common in HCC nodules, their presence highly supports malignancy diagnosis. Reduction of cytoplasmic volume (scant cytoplasm) has been introduced in previous reports as a diagnostic criterion for well differentiated HCC [9]. Eccentric nuclei has been reported in well differentiated HCC [10]. In our cases this finding was present in 35 % of HCC smears and none of cirrhotic nodules. Other rare criteria in cirrhotic nodules composed of large nucleoli (2 %), multiple nucleoli (6 %), high N/C ratio (4 %), irregular chromatin distribution (2 %), and broad cord (2 %) which was present in 50 %, 72.5 %, 87 %, 77 %, and 77.5 % of HCC nodules, respectively. So, according to our study these five criteria can be discriminatory between HCC and regenerative nodules. Pseudoacini formation (polygonal neoplastic hepatocytes surround cystically dilated canalicula) (1 %) and intra nuclear inclusion (4 %) were rare in regenerative nodules of cirrhosis, but also they were not common in HCC nodules (37.5 % and 20 %, respectively); thus, their presence can be in favor of HCC. Presence of atypical naked nuclei has been reported to be a very important criterion of HCC diagnosis [11]. Tendency to dissociation has been observed in highly well-differentiated HCC due to narrow cords [12]. In our study, the absence of atypical naked nuclei was highly in favor of regenerative nodule because it was present in 65 % of HCC nodules, and only 10 % of cirrhotic nodules showed this finding. In our study, nearly all of cirrhotic nodules showed abundant, monotonous and thick cytoplasm, but these criteria were also common in HCC nodules (32.5 %, 40 % and 40 % , respectively); therefore, these findings cannot discriminate between well differentiated HCC nodules in the background of cirrhosis and pure regenerative nodules of cirrhosis. Presence of bile duct epithelial cells in the smears of FNA cytology of the liver were seen in 68 % of cirrhotic and 45 % of HCC nodules, therefore it is not a good diagnostic criterion for HCC in the background of cirrhosis. In the previous reports it has also been present in 82 % and 45 % of the benign lesions and well differentiated HCC nodules, respectively [4]. Presence of spindle cells was also in favor of cirrhotic rather than HCC nodules (82 % vs. 19 %, respectively).

Our study emphasizes on the diagnosis of HCC vs. cirrhotic nodules by FNA, using a combination of criteria. Presence of thick, abundant and monotonous cytoplasm and the absence of broad cords, high N/C ratio, large nucleoli and multiple nucleoli are in favor of cirrhotic nodules. Presence of mitosis, transgressing endothelium, and eccentric nuclei are in favor of malignancy in the patients with cirrhosis, but their absence cannot exclude malignancy.
